# Effects of Depressive Symptoms and Family Satisfaction on Health Related Quality of Life: The Hong Kong FAMILY Study

**DOI:** 10.1371/journal.pone.0058436

**Published:** 2013-03-14

**Authors:** Hairong Nan, Paul H. Lee, Michael Y. Ni, Brandford H. Y. Chan, Tai-Hing Lam

**Affiliations:** Department of Community Medicine, School of Public Health, Li Ka Shing Faculty of Medicine, The University of Hong Kong, SAR China; Tehran University of Medical Sciences, Islamic Republic of Iran

## Abstract

**Objective:**

To examine the effect of depressive symptoms and satisfaction with family support (FS) on physical and mental Health Related Quality of Life (HRQoL).

**Methods:**

Data were obtained from the Hong Kong FAMILY Project baseline survey in 2009–2011, which included 16,039 community residents (age ≥20). The FS was measured using the Family Adaptation, Partnership, Growth, Affection, Resolve (APGAR, range 0–10) Questionnaire. HRQoL were assessed using the SF-12 version 2. Depressive symptoms were recorded using the Patient Health Questionnaire-9 (PHQ-9). Demographic and lifestyle variables, stressful life events, perceived neighborhood cohesion were also assessed.

**Results:**

In a multilevel regression model, socio-demographic and behavioral variables explained 21% and 19% of the variance in physical and mental HRQoL. The presence of depressive symptoms (PHQ-9 score ≥10, standardized coefficients, β of −1.73) and high FS (APGAR score 7–10, 1.15) were associated with mental HRQoL, after adjustment for age, education, household monthly income, drinking status, physical activity, chronic conditions, life stress and neighborhood cohesion. Not FS but the presence of depressive symptoms (β of −0.88) was associated with physical HRQoL. The presence of depressive symptoms in women than men were more associated with a poorer physical HRQoL (p<0.01) while depressive symptoms in men were associated with a decrease in mental HRQoL (p<0.001). The interaction between FS and depressive symptoms was nonsignificant in relation to HRQoL. Among those with depressive symptoms, high FS was associated with a better mental HRQoL (41.1 vs. 37.9, p<0.001) in women but not contribute to variance in men.

**Conclusions:**

Higher FS and presence of depressive symptoms were significantly associated with HRQoL in general population in Hong Kong. Among those with depressive symptoms, high FS was associated with a favorable mental HRQoL in women but not men.

## Introduction

Health Related Quality of Life (HRQoL) is a broad ranging concept affected in a complex way by the person’s physical health, psychological state, level of independence, social relationships, personal beliefs and their relationship to salient features of their environment [1]. Although no universal definition is available, HRQoL is increasingly recognized as a valid and reliable measurement in healthy populations and as an assessment of the effect of illness on well-being. In addition to the four major non-communicable diseases (NCD): cardiovascular diseases (48%), diabetes (4%), cancers (21%) and chronic respiratory diseases (12%) shared the leading causes of NCD deaths in 2008 [2], depressive symptoms is also the leading cause of years lost due to disability [3]. Chinese societies has undergone rapid urbanization and Westernization of lifestyles in recent decades, and has also witnessed rising life expectancy and an increasing burden of chronic disease [2]. Chronic conditions can induce a low level of HRQoL for the patients and also their family members. The presence of depressive symptoms and the simultaneous occurrence of chronic conditions had cumulative negative effects on HRQoL among community-dwelling older adults age 60 and above [4]. Identifying depressive symptoms among individuals with and without chronic conditions is therefore crucial for implementation of measures aimed at improving HRQoL in a community-dwelling population.

Studies have shown that positive social support emerges as a mitigating factor in chronic conditions both in the West [5], [6] and China [7]. Furthermore, it has been suggested that the family is the most important source of social support [8]. A number of studies, mainly cross-sectional have assessed the effect of family support (FS) on HRQoL in China. However, these studies are relatively small and focus only on minority and vulnerable groups, such as the chronically ill [9], [10] and the elderly [11], or were limited by the telephone interview method [12]. Among the few studies that examined the association prospectively, a positive social support was found to be associated with a more favorable HRQoL [13] and fewer uses of mental health services [14]. Nevertheless, these prospective studies may be limited by restriction to specific chronic conditions [13] or use of proxy indicators of mental health such as the utilization of health service [14]. FS plays an essential role in Chinese culture particularly in times of illness [15]. Hence, we aimed to examine the role of perceived satisfaction to FS in relation to HRQoL, among those with and without depressive symptoms. The effect of social relationships on mental health has been demonstrated on multiple levels, including family, work, and neighborhood [16]. Support at each level could mitigate the effect of depressive symptoms on quality of life, and thus we have examined this using multi-level analysis.

Studies have indicated that higher levels of depressive symptoms are associated with lower HRQoL in ill individuals and their family members. However, these findings were also limited by small and specific sample [17], [18], [19] or to certain conditions [20]. A cross-sectional survey in mainland China reported that FS and self-reported health status explained most of the variance in depressive symptoms [15]. Older women were more likely than men to be adversely affected by lack of FS and to report depressive symptoms [21]. Gender roles in Chinese societies are more differentiated than in the West [22], thus the hypothesis that women with adequate FS compared to men would be less affected by depressive symptoms in terms of HRQoL is tested in this community-dwelling sample.

In this study, we used a population-based household survey in Hong Kong to examine 1) the independent associations of depressive symptoms and adequate FS with HRQoL; 2) interaction between sex and depressive symptoms and interaction between sex and FS in relation to HRQoL; 3) FS moderate the effect of depressive symptoms on HRQoL and the interaction is sex-specific.

## Methods

### Ethics Statement

The study protocol was approved by the Institutional Review Board of the University of Hong Kong and all participants signed an informed consent form prior to the interview. For those aged below 18, a parental consent was also obtained from their parents.

### Study Sample

A population-based household survey entitled ‘FAMILY: a Jockey Club Initiative for a Harmonious Society’, was carried out from March 2009 to April 2011. Details of sampling and interview have been described elsewhere [23]. In brief, sampling was based on residential addresses provided by the Hong Kong Census and Statistics Department. First, residential addresses were randomly sampled in all of the 18 districts of Hong Kong, with sample sizes proportionate to the district populations. The random sampling was stratified by type of dwelling (e.g., public or private housing complexes). Before the survey began, a notification letter was sent to the selected households, explaining the purpose of the survey and assuring confidentiality. Trained interviewers then approached all selected residential addresses. We excluded those addresses in which the interviewers could not contact anyone after six visits, each at least one week apart. Each family member aged ≥15 years in the sampled household who could understand and respond to the interview in Cantonese was eligible and invited to participate. Family members were interviewed separately and privately, with data gathered in electronic format on laptop computers. Participants responded to a structured questionnaire under the supervision of the interviewer who was available to answer questions and clarify items. The present sampling scheme was chosen based on the objectives of the FAMILY Project, which is to recruit complete households in order to examine the relationships between all family members in terms of health, happiness and harmony treating household as a unit. We only retained households in which all family members agreed to participate.

### Socio-demographic Measures

Participants were asked whether they had any of the following major chronic conditions, as diagnosed by a physician: heart disease, stroke, diabetes, asthma, chronic obstructive lung disease, digestive diseases (gastric ulcer, hepatitis B or C, or cirrhosis), or cancer. The total number of those major chronic conditions was used in the present analysis. Height was measured with SECA 214 stadiometer (http://www.seca.com), with a precision of 1 mm. Weight was measured with Omron fat analyzer scale HBF-356 (http://www.omron-healthcare.com.sg), with precision at 0.1 kg for weight. Both height and weight were measured by trained interviewers with standard protocols [24]. Body mass index was defined as body weight divided by height squared (kg/m^2^). Socio-economic status was measured by educational attainment and household monthly income. Education was classified into two levels by the highest education attained: tertiary versus secondary and below. Household monthly income was classified into two levels: $20,000 and above and less than $20.000 (US$ 1 = HK$ 7.8). Participants were classified into high-risk drinking (engagement in binge drink in past one month and/or alcohol intake of ≥210 g (men) or ≥140 g (women) per week) versus low-risk drinking (including non-drinkers). Smoking behavior included current smoking (smoke at least one cigarette every day) and non-smoking (including ex-smokers). Involvement in physical activity was classified into two categories (less active versus more active) according to the International Physical Activity Questionnaire (IPAQ) [25]. ‘More active’ is defined as equates to approximately at least 1.5–2 hours of total activity per day, of at least moderate intensity activity. Demographic measures included age, gender, marital status and number of persons living in a household.

### Psychological Assessments

#### Physical and Mental HRQoL

The Medical Outcomes Study 12-item short-form version 2 (SF-12) [26] measures eight domains of health on 5-point Likert scales: Physical Functioning, Role Physical, Bodily Pain, General Health, Vitality, Social Functioning, Role Emotional, and Mental Health. Total scores for each domain are calculated and transformed into a 0 to 100 scale. The first four domains form the physical HRQoL scores and the latter four the mental HRQoL scores. These aggregated scores were converted into norm-based scores (mean, 50; standard deviation (SD), 10), where higher scores indicated a more favorable physical and psychological well-being [26].

The SF-12 version 2 has been validated in Chinese samples [27], [28]. Scores for the physical and mental components are calculated by the formula provided in the SF-12 user manual [29]. As these scores are not based on raw scores, Cronbach’s alpha cannot be calculated for the individual subscales. For our sample, the alpha for the 12-item scale was 0.86. The physical and mental HRQoL were weakly correlated (Spearman ρ = −0.14).

#### Satisfaction with family support (FS)

FS was measured using the 5-item Family Adaptation, Partnership, Growth, Affection, Resolve (APGAR) Questionnaire, which collects information on satisfaction with the aforementioned five areas of family function [30]. The 5 items were as follows: 1) I am satisfied that I can turn to my family for help when something is troubling me; 2) I am satisfied with the way my family talks over things with me and shares problems with me; 3) I am satisfied that my family accepts and supports my wishes to take on new activities or directions; 4) I am satisfied with the way my family expresses affection, and responds to my emotions, such as anger, sorrow, or love; and 5) I am satisfied with the way my family and I share time together. Scores range from 0 to 10, with higher scores indicating greater FS. The Chinese version has been used previously in Hong Kong with good reliability and construct validity in Hong Kong [31]. The internal consistency in the present sample was 0.94.

#### Perceived neighborhood cohesion

Perceived neighborhood cohesion was measured using 4 items, each rated on a 5-point Likert scale (scores range from 1 to 5), with higher scores indicating greater social cohesion [32]. For an example, one item asks, ‘people around here are willing to help their neighbors’. We omitted the fifth item (people in this neighborhood do not share the same values) from the original list because of its low factor loading (<0.40). The four items have been shown reliable in previous studies [33] and had an internal consistency of 0.86 in the present sample. These four items have been previously used in a local setting [34].

#### Depressive symptoms scores

The nine-item Patient Health Questionnaire (PHQ-9) was used to assess depressive symptoms [35], [36]. Participants rated the frequency of experiencing nine symptoms during the previous two weeks: 0) not at all, 1) on several days, 2) on more than half of the days, and 3) nearly every day. Scores range from 0 to 27, with higher scores indicating high severity of symptoms. A PHQ-9 score of 10 or higher indicates moderate or higher levels of severity. The reliability and validity of the Chinese PHQ-9 have been previously reported using pilot data from the present dataset [37]. The internal reliability in the present sample was 0.83.

#### Life stress

Participants were asked whether they have experienced any of 18 stressful life events, derived from the Recent Life Changes Questionnaire (RLCQ) [38], over the past 12 months. These life events include death of a family member or a close friend, unemployment, serious health problems, financial problem, changes in interpersonal relationship, and movement of residence. Four culturally relevant life events were added to the RLCQ list, including “I had a heavier workload (in the home, school or work settings)”, “somebody in my family had a serious health problem”, “a close friend had a serious health problem”, and “I set up a new household (e.g. moving out of parents’ home)”. About 30% of participants reported having experienced at least one stressful life event over the past 12 months. The top three most commonly experienced stressful life events were heavier workload (9.2%), worsening financial situation (6.5%) and serious health problem in oneself or family members (6.0%). Binary of stressful events (yes vs. no) was used in analysis.

### Statistical Analysis

Comparisons were made between the FAMILY Study participants and the 2011 Hong Kong general populations (Census and Statistics Department data) at both individual and household levels. Missing data on household income (N = 2214), education level (N = 84) were imputed using multiple imputation with additive regression, bootstrapping, and predictive mean matching by R package Hmisc (http://lib.stat.cmu.edu/S/Harrell/help/Hmisc/html/aregImpute.html). Results were presented as means with standard deviations (SD). Categorical data were analyzed using the χ^2^ test. Multilevel regression was used because more than one person could be interviewed in each household and thus their results would not be independent observations. Variables entered into the regression model included socio-demographic variables (age, sex, marital status, body mass index, number of chronic conditions, educational attainment, household income and neighborhood cohesion), and lifestyle variables (smoking, drinking and physical activity). Both beta (β) and standardized beta coefficients (computed by β times the SD of the corresponding independent variable) were reported to standardize the scales of different psychological assessments in regression analyses. To test hypothesis 2, the sex-depressive symptoms interaction and sex-FS interaction was fitted into a multilevel regression model that also included all other variables. In addition, the relationship between FS and depressive symptoms was examined separately for men and women. We divided participants into 4 groups by FS and severity level of depressive symptoms: those with high FS (APGAR 7–10) and without depressive symptoms (PHQ-9<10), those with low FS (APGAR 0–6) and without depressive symptoms, those with high FS and depressive symptoms (PHQ-9≥10), and those with low FS and depressive symptoms to estimate the mean physical and mental HRQoL scores in men and women separately. For all analyses, two-tailed p-values <0.05 were considered statistically significant. All statistical analyses were performed using IBM-SPSS Statistics software, version 19.0 and R statistical package.

## Results

### Sample Profile

A total of 7,864 households completed the interviews. The present sample represents 22.2% of the households originally approached as only complete households are included, where all eligible members agreed to participate. At the individual level, comparisons were made between the FAMILY Study participants and the 2011 Hong Kong general population (Census and Statistics Department data (http://www.census2011.gov.hk)) on age, gender and highest educational level attained. All dimensions showed only negligible differences, and the FAMILY Study sample was therefore judged to be representative of the Hong Kong general population [39]. At the household level, comparisons were made on household size and residential district which showed negligible differences. However, a medium-effect difference was found in type of housing (Table 1), where the FAMILY sample reported a higher proportion of individuals living in public housing compared to the census data. Data presented in this study are weighted by household income, household size (number of family members in a household) and housing type reported in the 2011 Hong Kong Population Census. A total of 16,039 respondents (7,312 men and 8,727 women, mean age of 47.1 (SD = 15.9)) were included in the present paper, but in the stratification analysis of physical and mental HRQoL only included 16, 025 with completed data.

**Table 1:** Representativeness of sample at household level (Number, %).

	FAMILY Project 2009–2011	C&SD population 2011	Effect size
Type of housing			0.35
Public rental housing	4,311 (54.8)	720,892 (30.4)	
Subsidised home ownership housing	1,335 (17.0)	377,615 (15.9)	
Private residential flats	2,153 (27.4)	1,242,982 (52.5)	
Other permanent housing	54 (0.7)	27,307 (1.2)	
Temporary housing	10 (0.1)	–	
Missing	1 (0.0)	–	
Number of persons in each household			0.06
1	1,688 (21.5)	404,088 (17.1)	
2	2,222 (28.3)	597,697 (25.2)	
3	1,818 (23.1)	575,316 (24.3)	
4	1,576 (20.0)	501,845 (21.2)	
5 or more	560 (7.1)	289,850 (12.2)	
Household monthly income (HK$)			
Below $2,000	541 (7.0)	85,394 (3.6)	0.19
$2,000–$3,999	942 (12.2)	129,332 (5.5)	
$4,000–$5,999	653 (8.4)	94,894 (4.0)	
$6,000–$7,999	663 (8.6)	121,173 (5.1)	
$8,000–$9,999	524 (6.8)	133,122 (5.6)	
$10,000–$14,999	1,385 (17.9)	297,830 (12.6)	
$15,000–$19,999	813 (10.5)	265,224 (11.2)	
$20,000–$24,999	752 (9.7)	235,695 (9.9)	
$25,000–$29,999	398 (5.1)	181,313 (7.7)	
$30,000–$39,999	424 (5.5)	269,283 (11.4)	
$40,000–$59,999	399 (5.1)	267,953 (11.3)	
$60,000–$79,999	125 (1.6)	117,260 (5.0)	
$80,000–$99,999	50 (0.6)	58,895 (2.5)	
$100,000 or above	79 (1.0)	111,428 (4.7)	
Residential district			0.06
Hong Kong Island			
Central and Western	142 (1.8)	89,529 (3.8)	
Wan Chai	47 (0.6)	54,887 (2.3)	
Eastern	564 (7.2)	194,249 (8.2)	
Southern	275 (3.5)	85,837 (3.6)	
Kowloon			
Yau Tsim Mong	288 (3.7)	112,986 (4.8)	
Sham Shui Po	571 (7.3)	134,795 (5.7)	
Kowloon City	410 (5.2)	124,218 (5.2)	
Wong Tai Sin	556 (7.1)	140,315 (5.9)	
Kwun Tong	792 (10.1)	214,300 (9.0)	
New Territories			
Kwai Tsing	864 (11.0)	168,553 (7.1)	
Tsuen Wan	381 (4.8)	102,570 (4.3)	
Tuen Mun	554 (7.0)	168,990 (7.1)	
Yuen Long	573 (7.3)	190,285 (8.0)	
Northern	363 (4.6)	99,453 (4.2)	
Tai Po	345 (4.4)	94,481 (4.0)	
Sha Tin	768 (9.8)	207,094 (8.7)	
Sai Kung	240 (3.1)	138,209 (5.8)	
Islands	131 (1.7)	47,611 (2.0)	

Cohen’s *w* effect size: small 0.1, medium 0.3, large 0.5.

Table 2 shows the characteristics of the sample. The overall mean score (SD) of physical and mental HRQoL was 49.6 (7.6) and 53.6 (7.8), respectively. Women were more likely than men to report depressive symptoms. Overall, 56.3% participants reported high FS (APGAR of 7–10), with a higher proportion of women reporting high FS compared to men (58.7% vs. 53.5%, χ^2^ = 44.2, p<0.001). Men were more likely to report higher physical and mental HRQoL, and higher perceived neighborhood cohesion compared to women. More men than women were current smokers or high-risk drinkers, and physically active.

**Table 2:** Characteristics of the study sample and its difference between men and women. ^a.^

Variables	Total (n = 16,039)	Men (n = 7,312)	Women (n = 8,727)	P values
Mean age in year (SD)^b^	47.1 (15.9) [20–103]	47.8 (16.0) [20–103]	46.5 (16.0) [20–100]	<0.001
Marital status (%)				<0.001
Never Married	22.2	22.2	22.1	
Married	70.1	74.3	66.5	
Widowed	4.8	1.6	7.6	
Divorced/Separated	2.9	1.9	3.8	
Education levels (%)				<0.001
Primary	23.5	19.5	26.6	
Secondary	49.3	49.1	48.9	
Tertiary or above	27.2	31.5	24.5	
Number of persons in each household				
1	5.9	–	–	
2	17.3	–	–	
3	25.1	–	–	
4	29.1	–	–	
≥5	22.5	–	–	
Household monthly income (HK$^c^, %)				<0.001
No income or <6 000	40.3	25.8	52.6	
6 000–9 999	15.6	15.4	15.7	
10 000–14 999	18.5	24.4	13.6	
15 000–19 999	7.6	9.7	5.8	
20 000–24 999	5.3	6.5	4.3	
25 000–29 999	2.7	3.8	1.8	
≥30 000	10.0	14.4	6.3	
Body mass index (kg/m^2^)	23.6 (3.7) [15.0–44.6]	24.2 (3.6) [15.0–44.6]	23.2 (3.8) [15.0–43.4]	<0.001
Number of chronic conditions d (%)				<0.001
0	69.5	69.1	69.9	
1	20.9	21.3	20.6	
2	7.3	7.3	7.2	
≥3	2.3	2.3	2.3	
Alcohol drinking status (yes, %)				<0.001
Non- or light drinker	97.5	95.9	98.9	
Current high risk drinker	2.5	4.1	1.1	
Smoking status (yes, %)				<0.001
Non- or ex-smoker	86.7	75.6	96.1	
Current smoker	13.3	24.4	3.9	
Physical activity (yes, %)				<0.001
Less active	64.6	62.7	66.3	
More active	35.4	37.3	33.7	
Health-Related Quality of Life (SF-12) e				
Physical component summary (PCS)	49.6 (7.6) [4.3–71.2]	50.5 (7.0) [4.3–71.2]	48.8 (8.1) [5.5–69.6]	<0.001
Physical Functioning	90.1 (22.7)	92.8 (19.9)	87.8 (24.6)	<0.001
Role Physical	90.1 (21.0)	92.0 (19.2)	88.5 (22.2)	<0.001
Bodily Pain	88.3 (21.5)	90.6 (19.7)	86.4 (22.8)	<0.001
General Health	46.5 (23.7)	48.5 (23.7)	44.8 (23.6)	<0.001
Mental component summary (MCS)	53.6 (7.8) [−0.5–79.3]	54.1 (7.4) [−0.5–76.1]	53.1 (8.2) [5.8–79.3]	<0.001
Vitality	69.8 (23.3)	71.9 (22.4)	68.1 (22.4)	<0.001
Social Functioning	91.2 (19.8)	92.4 (18.8)	90.1 (20.6)	<0.001
Role Emotional	89.7 (18.7)	91.2 (17.4)	88.4 (19.7)	<0.001
Mental Health	78.8 (16.9)	80.0 (16.1)	77.7 (17.5)	<0.001
Depression score (PHQ-9 score)	1.8 (2.8) [0–27]	1.5 (2.5) [0–27]	2.0 (2.9) [0–27]	<0.001
Level of depressive symptoms severity (PHQ-9 score, %)				<0.001
Minimal (0–4)	88.2	90.3	86.3	
Mild (5–9)	9.7	8.2	11.1	
Moderate (10–14)	1.5	1.0	1.9	
Moderately severe (15–19)	0.4	0.4	0.4	
Severe (> = 20)	0.2	0.1	0.3	
Life stress (yes, %)	30.7	29.7	31.6	<0.001
Satisfaction with family support (FS)	6.9 (3.2) [0–10]	6.7 (3.2) [0–10]	7.1 (3.2) [0–10]	<0.001
Level of FS (yes, %)				<0.001
High FS (APGAR 7–10)	56.3	53.5	58.7	
Low FS (APGAR 0–6)	43.7	46.5	41.3	
Neighborhood cohesion g	13.9 (2.4) [4]–[20]	13.7 (2.3) [4]–[20]	13.9 (2.5) [4]–[20]	<0.001

a P values for difference between men and women.

b Unless otherwise indicated, data presented are mean (standard division).

c US$ 1 = HK$7.8.

d Summed by self-reported heart disease, stroke, diabetes, asthma, chronic obstructive lung disease, gastrointestinal diseases, and cancer.

e Total scores for each domain range from 0 to 100.

f Measured by Family APGAR; higher scores indicate greater satisfaction with family support.

g Perceived neighborhood social cohesion; scores range from 1 to 5, higher scores indicate greater social cohesion.

### Socio-demographic Factors and Behaviors Associated with HRQoL

The multilevel regression model showed that socio-demographic variables explained 21% and 19% of the variance in physical and mental HRQoL (Table 3). The number of chronic conditions, age, presence of depressive symptoms (PHQ-9 score ≥10) and presence of life stress were found to be the four most significant factors associated with physical HRQoL (standardized coefficients (β) of −2.15, −2.26, −0.88 and −0.69, respectively, all p<0.001). The four most significant predictors of mental HRQoL were presence of depressive symptoms (β = −1.73), presence of life stress (β = −1.62), higher FS (β = 1.15), and age (β = 0.82) (all p<0.001). Lifestyle factors including high-risk drinking, physical activity were significantly associated with HRQoL (with exception of smoking for HRQoL). The significant role of aging in associations with both physical and mental HRQoL also maintained among those aged 60 and above participants. The FS-depressive symptoms interaction and the FS-sex interaction was not significant thus was omitted in the multilevel model of predicting HRQoL and physical HRQoL, respectively. The Intra-class correlation (ICC) of the physical and mental HRQoL model was 0.22 and 0.32, indicating small within-households correlation in HRQoL.

**Table 3:** The multilevel regression model of health related quality of life a with demographic variables, depressive symptoms and family satisfaction in the Chinese population aged 20 and above (N = 16,039).

	Physical HRQoL [0–100]				Mental HRQoL [0–100]			
Variables	Unstd. coefficient	Std. Error	Std. coefficient	t	Unstd.coefficient	Std. Error	Std. coefficient	t
Age (years)	−0.14***	0.004	−2.26	−25.56	0.05***	0.004	0.82	12.34
Sex (women vs. men)	−1.81***	0.10	−0.90	−5.39	−1.23***	0.16	−0.61	−7.56
Marital status (unmarried vs. married)	−1.09***	0.01	−0.50	−6.92	NA			
Number of chronic conditions	−2.83***	0.12	−2.15	−31.28	−0.66***	0.13	−0.50	−5.24
Body mass index (kg/m^2^)	−0.08**	0.02	−0.32	−2.89	0.06***	0.02	0.21	3.59
Education level (tertiarydegree vs. lower)	0.37*	0.14	0.16	2.41	0.43**	0.15	0.19	2.95
High risk drinking (yes vs. no)	1.07**	0.35	0.17	3.23	−0.75*	0.36	−0.12	−2.09
Physical activity (high vs. other)	1.05***	0.12	0.50	9.59	0.49***	0.12	0.24	4.14
Number of person in household	−0.35***	0.06	−0.49	−6.12	NA			
Household monthly income(20,000 vs. lower)	0.70***	0.15	0.34	4.99	NA			
Depressive symptoms(PHQ-9score ≥10)	−6.15***	0.47	−0.88	−12.76	−12.00***	0.49	−1.73	−24.57
Sex (male) by depressivesymptoms (PHQ-9 score ≥10)	2.20**	0.81	0.55	2.72	−3.77***	0.83	−0.94	−4.53
Family satisfaction (APGAR7–10 vs. 0–6)	NA				2.31***	0.16	1.15	14.68
Sex (male) by family satisfaction(low) interaction	NA				0.74***	0.22	0.64	3.39
Life stress (yes vs. no)	−1.51***	0.12	−0.69	−10.90	−3.52***	0.13	−1.62	−27.17
Neighourhood cohesion	0.06*	0.03	0.14	2.36	0.16***	0.03	0.39	6.12
R^2^ for the full model		0.21				0.19		
ICC b		0.22				0.32		

* p<0.05;

** p<0.01;

*** p<0.001.

a Physical and Mental health related quality of life were measured by SF-12 version 2.

b Intra-class correlation within family was 0.22 for Physical and 0.32 for Mental HRQoL.

FS was not associated with physical HRQoL, however neighborhood cohesion (β = 0.14, p<0.05) did weakly. Both higher FS and higher neighborhood cohesion were associated with more favorable mental HRQoL, after adjusting for age, sex, physical activity level, depressive symptoms, life stress and the number of chronic conditions. FS (β = 1.15), showed stronger association with mental HRQoL (z score = 13.2, p<0.001), compared to neighborhood cohesion (β = 0.39). Marital status, smoking, number of person in household and household income did not contribute any variance in mental HRQoL after adjustment for other variables.

### Differential Effect of Depressive Symptoms on Physical and Mental HRQoL between Men and Women

**Figure 1:**
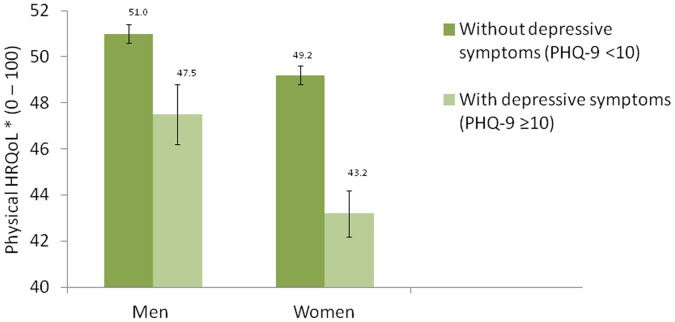
Physical health related quality of life (Physical HRQoL, bars) and their 95% confidence intervals in participants with depressive symptoms (PHQ-9≥10, 111 men and 228 women) and without depressive symptoms (PHQ-9<10, 7,196 men and 8,492 women) by sex. *Adjusting for age, education level, drinking status, physical activity level, body mass index, number of chronic diseases, life stress, neighborhood cohesion, household monthly income and the number of person in a household.

**Figure 2:**
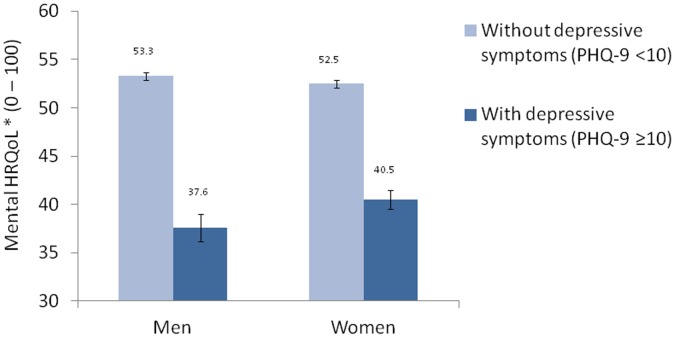
Mental health related quality of life (Mental HRQoL, bars) and their 95% confidence intervals in participants with depressive symptoms (PHQ-9≥10, 111 men and 228 women) and without depressive symptoms (PHQ-9<10, 7,196 men and 8,492 women) by sex. *Adjusting for age, education level, drinking status, physical activity level, body mass index, number of chronic diseases, life stress, and neighborhood cohesion.

Since the sex-depressive symptoms interaction was independently and significantly associated with both physical (β = 0.55, p<0.01) and mental HRQoL (β = −0.94 p<0.001), stratification for these two factors were conducted. Figure 1 shows that presence of depressive symptoms were associated with a significant drop of physical HRQoL levels in women than men (12% vs. 7%, p<0.01). On the other hand, the presence of depressive symptoms was associated with a decrease on mental HRQoL levels by 29% in men and 23% in women (p<0.001) (Figure 2).

### Effect of High FS on Mental HRQoL among Men and Women

Table 4 shows that both men and women with high FS (APGAR score 7–10) had higher mental HRQoL in comparison to their counterparts with low FS (APGAR score 0–6). Slight but significant FS-sex interaction (β = 0.64, p<0.001) was also observed in relation to mental HRQoL. High FS was associated with a 5% increase of mental HRQoL in women and a 4% in men (p<0.001). The interaction between FS and depressive symptoms was not significantly in relation to mental HRQoL. Nonetheless, when the participants were stratified further by sex, by depressive symptoms severity and by FS into 8 subgroups (Table 4), high FS was associated with a higher mental HRQoL in most of these groups, regardless of sex and depressive symptoms. Among men with depressive symptoms, high FS did not contribute variance of significance in mental HRQoL compared to those with low FS (36.2 vs. 38.1, p = 0.15).

**Table 4:** Mean (95% confidence interval, CI) and its difference of mental health related quality of life stratified by sex, satisfaction with family support (FS) and depressive symptoms.^a^

Mental HRQoL	N	Mean (95% CI)	Difference	P value*
Men with high FS	3,909	46.2 (45.4, 47.0)	0.9	0.07
Men with low FS	3,397	44.6 (43.9, 45.4)	−0.7	0.20
Women with high FS	5,121	47.6 (47.0, 48.2)	2.3	<0.001
Women with low FS	3,598	45.3 (44.7, 45.9)	Ref.	–
Men with PHQ-9≥10 and high FS	46	36.3 (34.4, 38.2)	−1.6	0.23
Men with PHQ-9≥10 and low FS	65	38.0 (36.1, 39.8)	Ref.	–
Women with PHQ-9≥10 and high FS	101	42.9 (41.5, 44.0)	4.7	<0.001
Women with PHQ-9≥10 and low FS	127	38.2 (36.8, 39.7)	Ref.	–
Men with PHQ-9<10 and high FS	3,863	54.1 (53.6, 54.6)	1.7	<0.001
Men with PHQ-9<10 and low FS	3,332	52.4 (51.8, 53.0)	Ref.	–
Women with PHQ-9<10 and high FS	5,020	53.6 (53.1, 54.1)	2.1	<0.001
Women with PHQ-9<10 and low FS	3,471	51.2 (50.6, 51.8)	Ref.	–

* P value for the difference in mental HRQoL between the reference group after adjusting for age, education level, drinking status, physical activity level, body mass index, number of chronic diseases, life stress, and neighborhood cohesion.

a Measured by the nine-item Patient Health Questionnaire (PHQ-9), ≥10 indicated moderate and above levels of depressive symptoms severity. High FS, family APGAR score 7–10; low FS, family APGAR score 0–6.

## Discussion

In a large representative sample of general population in Hong Kong Chinese, we found that low FS, presence of depressive symptoms (PHQ-9≥10) and presence of life stress were associated with unfavorable HRQoL, with the exception of FS having no association with physical HRQoL. We also found a significant interaction between sex-depressive symptoms and sex-FS for mental HRQoL. In contrast, only the sex-depressive symptoms interaction was significant for physical HRQoL. Regarding our third hypothesis, our study demonstrates that the interaction between FS and depressive symptoms was not significantly associated with physical and mental HRQoL. Nonetheless, among those with depressive symptoms (PHQ9≥10), high FS was associated with a favorable mental HRQoL in women but not men.

Both physical and mental HRQoL shared three common risk factors of depressive symptoms, life stress and older age, which is consistent with prior studies [4], [14], . Researchers have proposed that social support may buffer against the negative effects of stress on individuals’ well-being [41]. Associations of better FS with lower likelihood of reporting depressive symptoms [15], [21] and with favorable HRQoL have also been reported in previous studies [9], [14]. Nonetheless, our results have added to the understanding of the protective role of FS among people with depressive symptoms in a large group of community-dwelling individuals. We have also found that men are more likely to be affected by depressive symptoms than women as evidenced by the lower mental HRQoL. However, FS did not moderate the effect of depressive symptoms on mental HRQoL in men. Social support can be manifested at various levels and in this study, which has been measured at both the family and neighborhood levels, using FS and perceived neighborhood cohesion, respectively. FS, when compared to neighborhood cohesion, showed a stronger positive association with both physical and mental HRQoL. However, there are other sources of support which have not been included in this study, such as workplace and organization engagement, which may differentially affect HRQoL in men and women. Further studies are needed to explore how family, neighborhood, and other sources of support are linked to HRQoL among Chinese men and women.

Our study has addressed a vulnerable group in the community, namely, individuals with moderate or above depressive symptoms and lack FS are particularly prone to report low mental HRQoL. It would be worthwhile for primary care professionals or social workers to include a brief assessment of family function and support in routine consultations. For health promotion, enhancing family relationships could be an important primary prevention strategy to improve mental HRQoL in general and in high risk populations.

Experimental studies have demonstrated that physical activity could divert negative thoughts and elevate mood by enhancing secretion of endorphins [42]. Epidemiological studies have also reported notable associations between negative emotions, heavier body weight and physical inactivity [43], [44]. In line with previous studies, we found that physical activity showed a medium to strong positive association with physical (β = 0.50) and mental HRQoL (β = 0.24). Most of the unhealthy behavioural factors including high-risk drinking and inadequate physical activity were significantly associated with unfavourable HRQoL. These suggest that healthy lifestyle promotion may be recommended as one of the means to maintain well-being in Chinese societies.

The present study has three strengths. First, a large sample of households and individuals were randomly selected from all 18 districts in Hong Kong, which produced a representative sample of the general population. Second, this study has examined the effect of sex-depressive symptoms interaction and sex-FS interaction in relation to HRQoL in a community-dwelling population. Third, the study focused on examining the potential protective role of FS in a culture that emphasizes the importance of family ties. Given many cultural similarities shared between Hong Kong and other rapidly modernizing urban areas in China and other Asian nations, our Hong Kong results may be applicable across Asia. The present study has a number of limitations. First, the method of including only complete households in our sample lowered the response rate and could lead to a self-selection bias towards families with more satisfying relationships. To look for such a potential bias, we drew a subsample of households that did not achieve complete enrolment of every member and randomly selected one member from each such household to complete the survey (N = 1,930). A comparison analysis showed that the physical and mental HRQoL scores of these individuals did not differ to any substantial degree (Cohen’s d effect size <0.2) from the present study sample. Moreover, we did not find significant differences in FS comparing the complete households and incomplete households (mean of family APGAR 6.5 vs. 7.0, effect size = 0.145). Second, without access to medical records and biomarkers, we could not adequately assess the severity of the chronic conditions and the impact of disease control on HRQoL. The interpretation of our findings is limited by the cross-sectional design; however the second wave of the FAMILY project is due to be completed by May 2013. The prospective data will enable us to examine the interplay between depressive symptoms, perceived FS and lifestyle factors and to test whether baseline depressive symptoms and FS predict future HRQoL.

### Conclusion

Family support and presence of depressive symptoms was significantly associated with HRQoL in a representative sample of the general population in Hong Kong. Among participants with depressive symptoms, high family support also showed a significant protective role against deterioration in mental HRQoL for women but not men. For primary care professionals and social workers, family support assessment may be worthwhile in individuals with depressive symptoms.
